# Linking spontaneous speech synchronization and cognitive abilities: evidence from a syllable-timed language

**DOI:** 10.1007/s00426-026-02302-9

**Published:** 2026-04-25

**Authors:** María de Lourdes Noboa, Csaba Kertész, Neža Marija Slosar, Ferenc Honbolygó

**Affiliations:** 1 Doctoral School of Psychology, ELTE Eötvös Loránd University, Budapest, Hungary; 2 Institute of Psychology of the Faculty of Education and Psychology, ELTE Eötvös Loránd University, Izabella u. 46, Budapest, 1064 Hungary; 3 Brain Imaging Centre, Research Centre for Natural Sciences, Budapest, Hungary; 4 MEi:CogSci, University of Ljubljana, Ljubljana, Slovenia

## Abstract

Sensorimotor synchronization (SMS) refers to the coordination of audio and motor areas with external rhythmic stimuli, such as music or speech. The Speech-to-Speech Synchronization test (SSST) has been found to be a reliable measure of spontaneous synchronization to speech, and it has shown a bimodal distribution (high and low synchronizers) in stress-time and syllable-timed languages. Nonetheless it is unclear whether this pattern generalizes to other languages such as Hungarian, a syllable-timed language that possesses a fixed-stress pattern. This study aimed to replicate the bimodal distribution of the SSST in native Hungarian speakers and to explore associations between speech sensorimotor synchronization, finger tapping to music, musical background, and working memory. Thirty-one native Hungarian speakers completed the SSST (implicit version), a finger-tapping task, a working memory task (counting span), and a musical background questionnaire. We confirmed the bimodal distribution of SSST in our Hungarian sample. To examine the associations between SSST and other measures in covariate-adjusted analyses, we modeled SSST as a continuous variable to avoid information loss from dichotomization. We found that counting span was the only reliable positive predictor of SSST, whereas indices of tapping performance (consistency and asynchrony) and musical background (playing instrument(s), years of music education) showed no association. Despite limited sensitivity, these results support SSST as a cross-linguistic tendency and suggest that individual differences in spontaneous speech-to-speech synchrony relate more to working-memory capacity than to formal musical training. Future research should include neural correlates and explore how these individual differences manifest across different linguistic abilities.

**Supplementary Information:**

The online version contains supplementary material available at 10.1007/s00426-026-02302-9.

## Introduction

When experiencing music, humans are unable to resist clapping or tapping to the beat (Nettl, 2018; Tranchant et al., 2016). These are examples of sensorimotor synchronization (SMS) defined as the synchronization of our movements to an external rhythmic event, requiring the coordination between auditory and motor systems (Repp, 2005; Repp & Su, 2013). There is extensive literature on individuals’ SMS abilities to isochronous sequences and more complex rhythmic stimuli like music (Dalla Bella et al., 2017; Repp & Su, 2013; Scheurich et al., 2020).

However, spontaneous synchronization to speech has not been as studied as synchronization to music. This gap persists despite evidence of the activation of motor areas during speech perception (Wilson et al., 2004), and that neural oscillatory activity in auditory and speech-motor regions exhibits enhanced auditory-motor synchronization at 4.5 Hz. This frequency coincides with both the typical rate of mouth movements during speech production and the mean syllable rate across languages (Assaneo & Poeppel, 2018). Together, these findings suggest that speech, like music, may elicit spontaneous sensorimotor synchronization, yet this has been underexplored.

To address this gap, Assaneo and colleagues, (2019) developed the Speech-to-Speech Synchronization task (SSST). In this task, native English speakers listen to a trail of syllables, and at the same time, they whisper the syllable ‘tah’. By measuring the onsets of the heard and whispered syllables, it is possible to characterize the audio-motor synchronization either in an explicit (i.e., following an instruction to synchronize to the heard syllables with increasing rate) or implicit (i.e., without any instructions to do so) situation. The degree of audio-motor synchronization has been reported to exhibit a bimodal distribution, with descriptive labels of “high” and “low” synchronizers.

High synchronizers were participants who successfully synchronized their produced speech to the perceived external speech while the low synchronizers were participants who did not. In the implicit version of the task, participants were not instructed to synchronize, and therefore in the high synchronizers group the auditory-motor synchronization occurred spontaneously. Previous studies have identified a preferred “speech rhythm” between 2 and 8 Hz for auditory-motor synchronization (Assaneo & Poeppel, 2018; Poeppel & Assaneo, 2020). Because the stimuli in the SSST had a consistent syllable rate of 4.5 Hz, high synchronizers may be able to capitalize on this preferred rhythm, whereas low synchronizers may not.

Previous studies suggest that high and low synchronizers may differ across cognitive domains. For example, high synchronizers exhibit greater sensitivity to faster speech rates, indicating a broader optimal temporal processing range than low synchronizers, whose performance declines at higher rates (Kern et al., 2021). Additionally, the neural responses of high synchronizers showed an enhanced synchronization to the stimuli’s patterns of energy change overtime (envelope) and an enhanced white matter volume in frontal-auditory pathways (Assaneo et al., 2019). The authors suggest that these functional and structural brain differences facilitate auditory-motor synchronization.

Musical background has been studied as predictor of group affiliation of high and low synchronizers. It has been found that high synchronizers had more years of musical background (Assaneo et al., 2019; Kern et al., 2021) and that musical training was positively correlated with the degree of synchrony (Rimmele et al., 2022). It is suggested that musical training enhances fine precise auditory skills and therefore aids in speech processing (Patel, 2012; Tierney & Kraus, 2014). Nonetheless, other studies have found no associations between musical training and group membership (Sjuls et al., 2023). Moreover, differences in temporal sensitivity between groups could not be fully explained by musical training alone, suggesting that additional cognitive or perceptual factors may contribute to individual differences in audio-motor synchronization (Kern et al., 2021).

Therefore, it remains important to examine how musical background and cognitive factors, such as working memory, relate to individual differences in SSST performance and other synchronization measures. Working memory has been suggested to support speech processing on the maintenance and integration of linguistic information over time: participants with stronger audio-motor synchronization and higher working memory capacity had better speech comprehensive performance, especially in faster speech rates where comprehension becomes more demanding (Lubinus et al., 2023).

Working memory has also been positively linked to rhythm production in music (Frischen et al., 2022; Kim et al., 2023). It has been suggested that working memory supports temporal information processing by encoding and maintaining the temporal intervals to be used later as a reference (Broadway & Engle, 2011; Gibbon et al., 1984; Teki & Griffiths, 2014). When presented with a tapping to music task, participants have been shown to deploy different strategies to solve the task, for instance, creating a subdivision of a given interval, a strategy that relies on working memory capacity (Repp & Doggett, 2007).

An unexplored aspect is how individual differences influence the relationship between degree of synchrony in the SSST and SMS to music, measured traditionally with the finger-tapping paradigm. If a higher degree of synchronization to speech can be predicted from tapping performance, there might be a common mechanism that supports SMS skills. SMS requires precise temporal integration between auditory and motor cortices to adequately extract and predict a sound’s onset, as well as to discriminate small timing variations which is fundamental not only for music but also for speech processing (Tierney & Kraus, 2014), therefore precise auditory timing can be one of the proposed mechanisms that supports rhythm processing in music and speech (Fiveash et al., 2021).

Fiveash and colleagues (2021) also highlight that this precise neural timing might be supported by entrainment of neural oscillations. As noted above, neural oscillations seem to have a preferred “speech rhythm” around 4.5 Hz (Assaneo & Poeppel, 2018; Poeppel & Assaneo, 2020). Likewise, apart from an auditory-motor entrainment found in high synchronizers, motor-auditory entrainment has also been found, suggesting that there are top-down effects from the motor system to enhance perception (Assaneo et al., 2020), and the entrainment of neural oscillations might be the mechanism that supports the bidirectional audio-motor coupling.

Another interesting aspect of the bimodal distribution of SSST is whether spontaneous synchronization to speech reflects language-specific rhythmic properties or a broader mechanism supporting speech processing. The bimodal distribution has been found in other Germanic languages that follow a stress-timed linguistic rhythm such as German (Assaneo et al., 2020) and Norwegian (Sjuls et al., 2023). More recently the bimodal distribution has also been found in syllable-timed languages, such as Mandarin (Zhu et al., 2024) and Spanish (Gómez Varela et al., 2024).

The present study had two aims. First, to examine whether this bimodal pattern of SSST extends to Hungarian speakers. Hungarian belongs to the Uralic language family (Laakso, 2011), often characterized as a syllable-time language (Varga, 2002), with a fixed-stress pattern, and stress is predictably assigned to the first syllable (trochaic stress), independently of the type of word (simple, derived, or compound, Vogel, 2007), making it an informative case for assessing potential crosslinguistic regularities in spontaneous speech synchronization.

The second aim was to test whether individual differences in SSST degree are associated with tapping performance (consistency, asynchrony), musical background (playing instrument(s), years of music education) and working memory (counting span). Based on proposed shared mechanisms for rhythm processing in music and speech (Fiveash et al., 2021; Tierney & Kraus, 2014) and links between working memory and speech and rhythm processing (Frischen et al., 2022; Kim et al., 2023; Kraus et al., 2012; Lubinus et al., 2023) as reviewed above, we predicted that tapping performance and working memory and musical background would be all significant predictors.

## Materials and methods

### Participants

Thirty-four healthy young adults (*M*_*age*_ = 20.7 years; *SD*_*age*_ = 2.9; 5 males, 29 females) participated in this study. All participants were right-handed, had normal or corrected-to-normal vision, and had normal hearing levels as confirmed by the audiometry measurement. Participants were native Hungarian speakers and provided written consent. Most of them were undergraduate students who received course credit for participation. None reported developmental, learning, or language disorders. The experiment was approved by the United Ethical Review Committee for Research in Psychology (EPKEB), Budapest, Hungary, and was conducted following the Declaration of Helsinki. Three participants were excluded (2 females and 1 male) due to incongruent trials in the SSST (see Sect. 3.1), resulting in a final sample of thirty-one participants.

### Tasks and measures

#### Speech-to-Speech Synchronization Test (SSST)

We employed the implicit fixed version of the SSST Assaneo and colleagues, (2019), which we adapted to the Hungarian language. In this version, participants were not instructed to synchronize their speech with the auditory stimuli; therefore, the obtained degree of synchrony is intended to index spontaneous synchronization of their produced speech to the perceived stimuli.

Stimuli consisted of Hungarian syllables synthesized using PROFIVOX waveform speech synthesizer (version 2.6, DTT BUTE) with a female voice (Eszter). All phonemes were equal in pitch (200 Hz) and duration (111 ms) matching the original English stimuli rate of 4.5 syllables/s (Lizcano-Cortés et al., 2022). Stimuli were presented binaurally via headphones at a mean sound pressure of 70 dB. The twelve syllables (see Supplementary Material) were randomly combined without gaps or consecutive repetition, forming a 60 s sequence. The experiment was implemented in MATLAB Version: 9.13.0 (R2022b) using an adapted version of the original script provided by the SSST authors with instructions translated into Hungarian language (Assaneo et al., 2019).

**Fig. 1:**
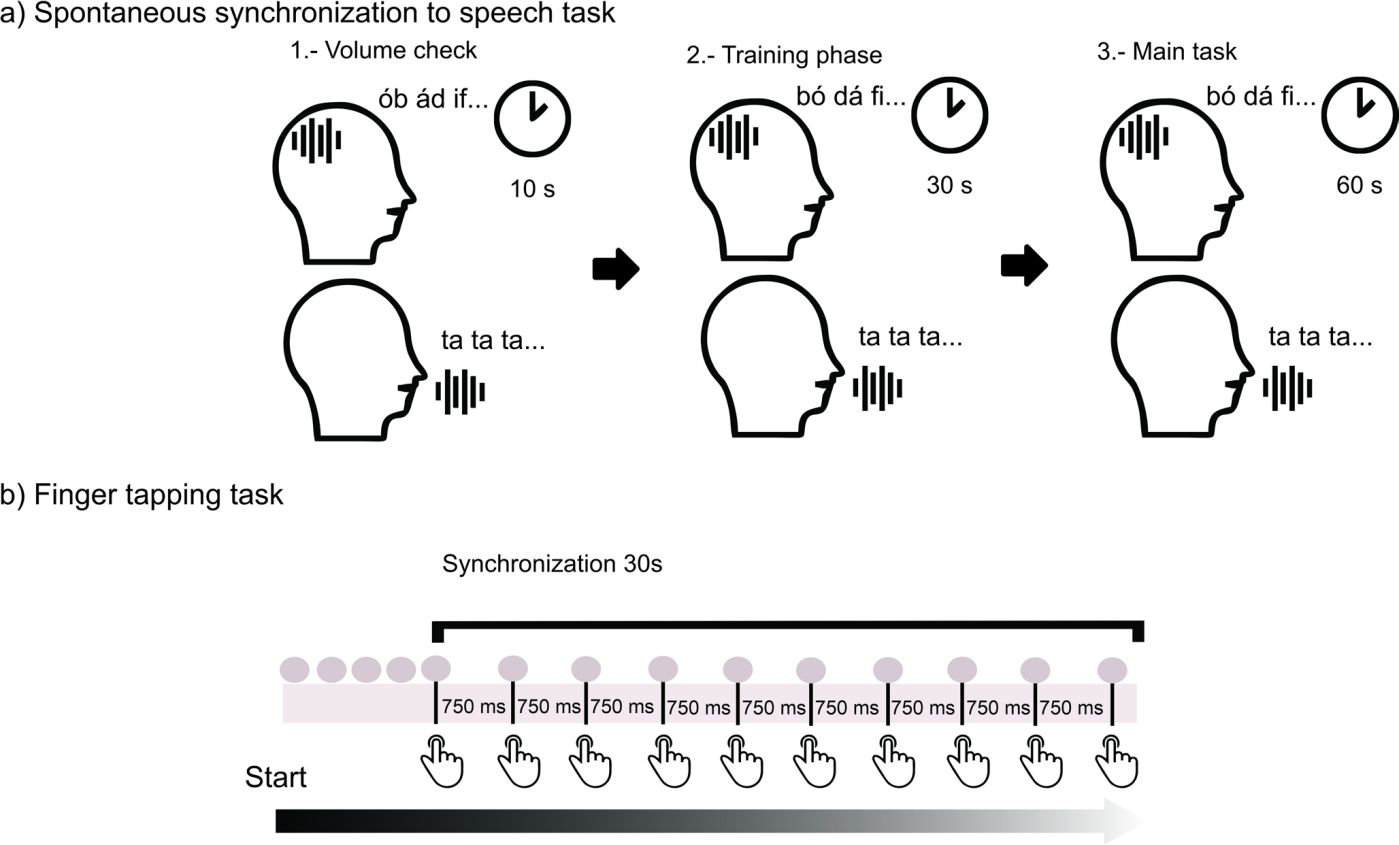
Graphic representation of the sensorimotor synchronization to speech and music tasks. **a**) Speech-to-Speech Synchronization task (SSST, implicit version): the task consisted of three phases. First, during the volume check, participants listened to a trial of reversed concatenated syllables while whispering “ta” to verify they could not her themselves. Second, in the training phase, participants listened to a continuous repetition of the syllable “ta” for 10 s and then practiced whispering “ta” at the same pace of the sequence for two 10-s training trials. Finally, in the main task, participants completed two 60-s trials in which they whispered “ta” while listening to the Hungarian syllable trail (**b**) Finger-Tapping to Music Task: Participants tapped to the beat of either a metronome or an instrumental version of a song at 80, 120, or 150 bpm; each trial lasted 30 s (three trials per condition). A brief spontaneous motor tempo familiarization preceded the main tapping trials

The procedure of the SSST (see Fig. 1a) included:

- Volume check: Participants whispered “ta” while listening to reversed syllable sequences. The audio volume was set to ensure they could not hear their voices. After they had confirmed this, they were told to proceed with the training phase.
- Training phase: Participants were presented two trials in which they listened to a continuous repetition of the syllable “ta” for 10s. Then syllable sequences were presented at 4.5 Hz, and participants were instructed to whisper “ta” at the same pace of the syllable sequence for 10 s.
- Main task: Participants were presented two 60 s trials in which they listened to the syllable sequence while continuously whispering “ta” and fixating on a central cross. To ensure participants remained attentive, each trial began with an instruction to focus on the syllables they heard, as they would later be asked to identify some of them. Following each trial, participants responded to four yes/no questions regarding the occurrence of specific syllables. (Assaneo et al., 2019; Lizcano-Cortés et al., 2022).

#### Tapping task to metronome and music

The finger-tapping task followed Kertész and Honbolygó (2021), and was implemented using an AKAI MK3 MIDI controller (see Fig. 1b). Participants tapped with their dominant hand while listening to either metronome sounds or music through headphones connected to a Steinberg U-22 interface. Playback and recording were managed with Cubase Elements 12 (version 12.0.40, 2022).

- Metronome condition: Consisted of three 30 s trials in different tempi (80, 120, 150 bpm), using a woodblock sample from the Cubase5 library.
- Music condition: Consisted of three 30 s instrumental versions of three popular songs, Dream, dream, dream (Everly Brothers) − 80 bpm, Michelle (The Beatles) − 120 bpm, and Johnny B Goode (Chuck Berry) − 150 bpm. The songs were rendered from MIDI scores using virtual instruments with the same instrumentation to avoid timbral differences. The vocal melody and characteristic instrumental parts (e.g., guitar riffs) were removed to avoid the advantages of song familiarity, leaving simple piano chord accompaniments with a double bass bassline and drumbeats.

Participants received instructions and a brief demonstration before data collection. If a participant misunderstood the task, adopted an atypical strategy (e.g., tapping in antiphase or double time), or tapping too softly for detection, the procedure was repeated after clarification. To familiarize participants with the MIDI controller, they first completed a 30-second spontaneous tapping trial (spontaneous motor tempo, SMT).

The main task consisted of six trials. Three trials for the metronome condition and three for the music condition, each presented at three different tempi (80, 120, 150 bpm). Participants were instructed to tap at the same tempo as the stimuli for approximately 30 s. Synchronization accuracy and tapping consistency were calculated in the statistical analysis to measure tapping performance (Kertész & Honbolygó, 2021).

#### Counting span

Working memory was assessed using a computerized counting span task presented in Presentation software (v. 21.1, Neurobehavioral Systems). Participants were asked to count aloud the blue circles on the computer screen, while ignoring distractors (yellow circles and blue squares). They were required to maintain the final counts of blue circles in each sequence until the end of each block. After each block (2 to 6 pictures per sequence), they were required to recall all numbers. A short demonstration was done by the experimenter, and participants were assured that there was no time limit for counting. The task consisted of three blocks with five sequences of stimulus sets (2,3,4,5,6) in each. The participant’s counting span for each block was calculated as the highest stimulus set size that was recalled in the correct order(Virag et al., 2015). To obtain the final counting span for each participant, we calculated the mean performance across the three blocks. The administration and completion of this task took approximately ten minutes.

#### Musical background questionnaire

Participants completed a questionnaire assessing formal musical education. Lacking a standardized questionnaire on the musical background in Hungarian, we asked questions about whether they had received music education, the age of onset, which instrument(s) they play(ed), and the total years of musical education. The mean years of musical education reported was 3.90 years, *SD* = 4.70 years. The mean number of instruments played was 1.07, *SD* = 1.15. Of our participants, 19 reported to have received musical education, while 12 reported not having received musical education. Lastly, 7 of the participants reported being active musicians, while the rest (*N* = 24) reported not being active.

### Procedure

The experiment session began with participants filling out the consent form, Edinburgh Handedness questionnaire, and musical background questionnaire. Afterward, participants went inside a sound isolation booth where they took the audiometry test to confirm normal audition levels. Participants were seated in front of a PC and had a microphone placed close to their mouths. The SSST, tapping task, and counting span were done in a pseudo-random order. After each task, the experimenter would go inside the room to remove or adjust equipment and explain to the participants the instructions for the following task. The whole experimental session lasted approximately forty minutes.

## Data analysis

### SSST

The goal of this analysis was to obtain the degree of synchronization between perceived (trail of random syllables) and produced signals (whispered ‘ta’). We followed the procedure described in Assaneo et al. (2019) and Lizcano-Cortés et al. (2022), using the MATLAB script provided by the authors. First, we inspected the audio outputs for each participant and applied the exclusion criteria from Lizcano-Cortés et al. (2022). Two participants were excluded due to silence gaps longer than 3 s, confirmed by the lack of congruency between their two trial outputs.

To obtain the degree of synchronization, we calculated the envelope of each signal, and we applied a filter around the syllabic rate to the produced signal. Subsequently, the phase-locking values (PLVs) between the produced and perceived filtered envelopes were computed within windows of 5 s in length, with an overlap of 2 s. The PLVs for each time window within each output audio file were averaged, obtaining one PLV per run.

To ensure consistency between runs, a fitted linear regression was applied with the PLV from the first run as the independent variable and the PLV from the second run as a dependent variable. If a participant had a pair of PLVs outside the 95% confidence intervals of the fitted, they would be labeled as “incongruent” and excluded (*n* = 3). For the remaining participants, the degree of synchrony was calculated from the PLV averaged across runs. This value provided the probability of the participant being identified as either a high or low synchronizer.

We applied a density estimation via Gaussian finite mixture modeling to visualize the distribution of the degree of synchronization of each participant, as it has done in previous studies (Assaneo et al., 2019; Sjuls et al., 2023; Zhu et al., 2024, 2025). The analysis was done with the R statistical software’s ‘mclust’ package (v4.3.1 R Core Team 2023).

### Tapping task

For each trial in both conditions (metronome and music), we calculated two measures: synchronization accuracy and tapping consistency as described in Kertész and Honbolygó (2021). The first ten taps of each trial were excluded from the analysis. We used Rayleigh’s test to remove trials where tapping did not significantly differ from a random distribution, and we marked those trials with NaN excluding them from averaging. Outliers in inter-tap intervals (ITIs) were identified by collecting ITIs for all participants and removing those greater than the third quartile plus three times the interquartile range (Q3 + 3*IQR) or smaller than the first quartile minus three times the interquartile range (Q1–3IQR).

Two data analysis methods were used to calculate the difference between each tap and the nearest target beat. To measure tapping accuracy, the mean of the absolute values of differences was divided by the tempo of the music, resulting in a variable that shows the deviation from the reference as a percentage. A value of 0 indicates total synchrony with the stimulus.

To calculate tapping consistency, circular statistics were applied (Falk et al., 2015). A resultant vector (R) is calculated from all taps in a single trial, where the R vector’s length showed a participant’s tapping consistency, a hypothetical value of 1 meaning total consistency, and 0 total inconsistency. The individual tapping asynchronies were converted into points on a unit circle, with each point representing the distance from the nearest target beat. A value of 0 degrees on the circle indicates a perfectly timed tap.

For each participant, we obtained six values (two stimulus types x three tempi: 80, 120, 150 bpm). These were averaged across trials by stimulus type and tempo. The resulting mean consistency and accuracy scores were later used for further statistical analysis resembling the approaches of Woodruff Carr et al. (2014) and Politimou et al. (2019).

### Statistical analysis

Statistical analysis was done with R (v4.3.1 R Core Team 2023). The SSST distribution in this sample was bimodal (“Low” vs. “High” synchronizers), and we report this descriptive characterization. We ran a multiple linear regression model to predict the degree of synchrony (SSST), bounded 0–1. Consistent with our focus on individual differences, we analyzed SSST degree as a continuous bounded outcome to preserve information, avoid threshold misclassification near the cut-point, and reduce the loss of precision and power that dichotomization creates considerations that are especially important with a small sample (MacCallum et al., 2002).

Predictors included tapping performance, musical background, and working memory, as follows: tapping consistency (higher = better), tapping asynchrony (lower = better), playing instrument(s) (0 = no instrument, 1 = ≥ 1 instrument), years of music education, and counting span. Continuous predictors were standardized (z-score transformed) to facilitate interpretability of model coefficients; the binary predictor was kept as 0/1. Analyses used complete cases only (no imputation). All tests were two-sided (α = 0.05), and we report point estimates with 95% CIs. The regression equation was the following:

$$\begin{array}{c}SSSTdegreesync\sim\:consistency\left(z\right)+\:asynchrony\left(z\right)+\\instrument\left(0/1\right)+years\left(z\right)+counting\left(z\right)\end{array}$$

Model assumptions were assessed using the Shapiro-Wilk test for normality of residuals and the Breusch-Pagan test for homoscedasticity. Multicollinearity was evaluated through Variance inflation factors (VIFs). If any assumptions were violated, a robust MM-estimator regression (down-weighting outliers) as a confirmatory alternative was planned.

To quantify each predictor’s unique explanatory value, we computed partial *R*^2^ from nested Ordinary Least Squares (OLS) models. Given the sample size (*N* = 31) and five predictors, we prioritized effect estimates and confidence intervals over dichotomous decisions and avoided over-fitting (no stepwise selection; no higher-order terms). We did not report post-hoc power; instead, we conducted a sensitivity analysis showing that, for a five-predictor model at this sample size, power is low for small unique effects (e.g., $\:{f}^{2}\approx\:\:.05$) and becomes adequate only for moderate-to-large effects. This approach helps prevent over-interpretation of null findings while transparently communicating what magnitudes the design could detect.

## Results

### Bimodal distribution of the SSST in the Hungarian population

**Fig. 2:**
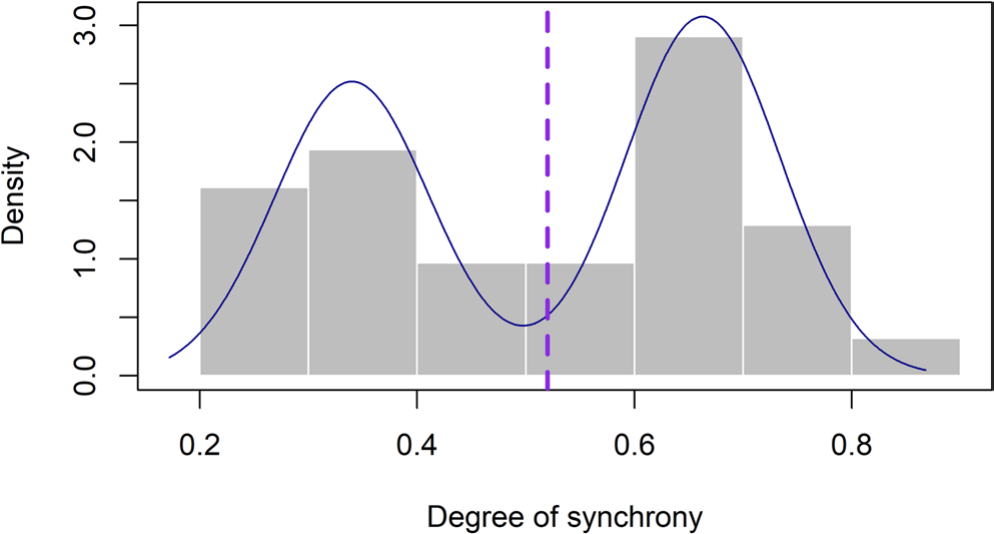
Distribution of the Speech-to-Speech Synchronization task (SSST) in native Hungarian speakers. The histogram shows the degree of synchrony (SSST) averaged across the two trials. The blue lines represent the fitted normal distribution of the two components identified with a model-based cluster analysis. The first component was labeled as low synchronizers (M = 0.340, SD = 0.075), and the second component as high synchronizers (M = 0.663, SD = 0.071). The dotted vertical line marks the sample mean (0.52) for visualization only; while values at or above were labeled “high” and values below “low,” these labels refer solely to mixture‑model components for comparability with prior literature, with all inferential analyses conducted on the continuous SSST measure

We observed a bimodal-like distribution of the degree of synchrony in our Hungarian sample (see Fig. 2). A Shapiro-Wilk test showed a significant departure from normality, W(31) = 0.93, *p* =.037. Model-based cluster analysis identified the best fitting model with 2 components (BIC = 20.275). As a result, we identified 14 low synchronizers (*M* = 0.340, *SD* = 0.075), and 17 high synchronizers (*M* = 0.663, *SD* = 0.071). The observed pattern is broadly consistent with previous reports that have shown a tendency to have a higher number of high synchronizers (Assaneo et al., 2019; Lizcano-Cortés et al., 2022). All subsequent analyses used the continuous SSST degree.

### Predicting Spontaneous Speech Synchronization (SSST) from tapping performance, musical background and counting span

Multiple linear regression examined associations between SSST and five predictors: tapping consistency (z), tapping asynchrony (z), playing instrument (0/1), years of music education (z), and counting span (z). The overall model was not significant, F(5,25) = 1.546, *p* =.212, R² = 0.236. Counting span was the only significant predictor (B = 0.080, *p* =.029), while all other predictors showed non-significant associations: tapping consistency (B = − 0.115, *p* =.062), tapping asynchrony (B = − 0.085, *p* =.130), playing instrument (B = 0.126, *p* =.210), and years of music education (B = − 0.032, *p* =.485).

Model diagnostics confirmed OLS assumptions: residuals were approximately normal (Shapiro–Wilk *p* =.282), homoscedastic (Breusch–Pagan *p* =.687), and multicollinearity was acceptable (all VIFs ≤ 3.56).

## Discussion

Our study had two aims: First, to replicate the bimodal distribution of the implicit version of the Speech-to-Speech Synchronization Test (SSST), to measure the spontaneous auditory-motor synchronization to speech in a Hungarian population; Second, to reveal the commonalities between SMS to speech and music, as well as to identify if other variables such as musical background and working memory are associated to SSST performance. Participants completed a finger-tapping task to metronome and music, and a counting span task. We observed a bimodal-like SSST distribution in our Hungarian sample, consistent with prior studies. Multiple regression revealed that counting span significantly predicted SSST; however, the overall model was not significant. Tapping consistency showed a small non-significant negative trend while tapping asynchrony, musical background (number of musical instruments and years of musical education) were not significant predictors.

### Bimodal distribution in Hungarian population

Our results corroborate the previously reported bimodal distribution observed in stress-timed languages such as English (Assaneo et al., 2019), German (Assaneo et al., 2020) and Norwegian (Sjuls et al., 2023), as well as syllable-time languages such as Spanish (Gómez Varela et al., 2024) and Mandarin (Zhu et al., 2024). We used the implicit version of the SSST where participants were not instructed to synchronize their produced speech to the perceived external speech. Nevertheless, a subset of participants, the high synchronizers, spontaneously aligned their onset of the whispered syllable “ta” to the heard syllable trail, indicating an enhanced audio-motor coupling in the absence of explicit instruction (Assaneo et al., 2019).

We administered the task in Hungarian, a language often characterized as syllable‑timed and with a stress-fixed pattern, in which the accent falls on the first syllable, resulting in a distinctive rhythmic profile (Varga, 2002). Our findings align with previous results and suggest that the SSST measures a spontaneous cross-linguistic tendency, which has been consistently reported across languages of different rhythmic qualities.

In the SSST, the stimuli were presented at the constant rate of 4.5 Hz, often described as a natural speech rhythm approximating the average mean syllable rate (Assaneo & Poeppel, 2018). Evidence suggests that auditory-motor coupling is particularly enhanced within this frequency range. At both neural and behavioral levels, low-frequency oscillations show enhanced sensitivity to rhythms around ~ 4–5 Hz, and their entrainment to these preferred rates may facilitate SMS (Assaneo et al., 2019; Assaneo & Poeppel, 2018). Neural entrainment, SMS and precise auditory timing have been proposed to support rhythm processing in music and speech (Fiveash et al., 2021). Importantly, stronger SMS to speech has been associated with better speech comprehension performance, particularly at faster speech rates (Lubinus et al., 2023) as well as enhanced syllable discrimination abilities (Assaneo et al., 2020).

Taken together prior work is consistent with the view that SMS to speech may support speech processing and suggest the presence of a cross-linguistic tendency of spontaneous synchronization of produced and perceived external speech in the general population. However, it remains unclear how SMS to speech and this specific spontaneous tendency may be associated with the processing of other rhythmic stimuli such as music.

### Tapping performance to music and spontaneous speech to speech synchronization

We aimed to test commonalities between speech and music by asking whether performance in a finger-tapping task predicts the degree of synchrony (SSST). We expected positive associations for tapping consistency and negative associations for asynchrony. However, in the covariate-adjusted model neither tapping measure emerged as a significant predictor. Consistency showed a weak negative trend, which should be interpreted cautiously given the small sample size and the overlap between tapping indices, and may reflect residual multicollinearity or task features rather than a stable association.

One possible explanation for the lack of significant findings is that the SSST and the tapping task may measure different mechanisms of synchronization. Both the SSST and the tapping task require auditory motor coupling. The SSST requires auditory-motor coupling to align articulatory movements to the perceived speech rhythm. On the other hand, in the tapping task, the auditory-motor coupling is required to synchronize the finger taps with a regular, isochronous beat. This process relies on building temporal predictions to anticipate the next beat and likely entails greater motor control (Repp, 2005; Repp & Su, 2013). For this reason, other cognitive factors, such as working memory, could contribute to SMS performance in music as has been previously shown (Colley et al., 2018; Kim et al., 2023; Noboa et al., 2025).

Additionally, the tapping task employed three tempi: 80, 120, and 150 bpm (corresponding to 1.33, 2.00, and 2.50 Hz) with the protocol centered on 120 bpm (2 Hz), a rate close to the preferred tempo for spontaneous synchronization in general population in musical contexts (McAuley et al., 2006; Moelants, 2002, 2003). In contrast, the SSST measures spontaneous speech-to-speech synchrony at 4.5 Hz, corresponding to the mean syllabic range (Assaneo & Poeppel, 2018). This discrepancy in temporal scales may attenuate cross-task associations: finger-tapping entrainment typically peaks near 2 Hz (Repp, 2005; Repp & Su, 2013), whereas speech rhythm unfolds near 4.5 Hz, and tapping at substantially higher rates (≈ 4–5 Hz) approaches biomechanical limits (ceiling ~ 6 Hz, Shourijeh et al., 2017). Furthermore, our procedure averaged consistency and asynchrony across tempi and stimuli to obtain more robust indicators (Lê et al., 2020), which may also obscure tempo-specific relationships (e.g., an association present at 120 bpm but absent at 80 or 150 bpm).

In the context of musical rhythm processing it has been previously suggested that rhythm processing skills are multidimensional, the performance of rhythmic tasks can be dissociated for instance in rhythm perception vs. rhythm production (Dalla Bella et al., 2024; Fiveash et al., 2022), performance can also vary depending on the characteristics of the stimuli (Tierney & Kraus, 2015), and the type of prediction mechanisms that stimuli can elicit, for instance beat-based predictions are elicited by isochronous and highly regular stimuli while memory-based predictions are elicited by learning the associations between events (Bouwer et al., 2020).

Accordingly, when comparing SMS to speech rhythm versus musical rhythms, dissociations in task performance may emerge. Despite commonalities in music and speech rhythm processing, cognitive and motor demands may differ; therefore, participants may rely on different synchronization and prediction strategies when engaging with speech versus music related tasks.

Consequently, the observed lack of correlation between SSST and tapping measures should be interpreted cautiously, given these cross-frequency and averaging constraints. Future research should compute tempo-specific correlations and models, and adopt rate-matched paradigms (e.g., speech-paced tapping or alternative motor tasks) to better assess cross-domain coupling. Nonetheless, the present findings suggest that rhythmic synchronization in speech and music may rely on domain-specific mechanisms.

### Musical background and working memory in sensorimotor synchronization

Lastly, we also examined whether musical background and working memory were associated with individual differences in degree of synchronization from the SSST. Counting span emerged as the only reliable positive predictor of SSST, whereas tapping consistency showed only a borderline negative association, and asynchrony, playing musical instrument(s), and years of music education were not significant.

These findings suggest that working memory capacity may support audio-motor coupling. It has been found that working memory can modulate the detection and regulation of feedback errors during speech (Guo et al., 2017). In the context of SSST, constant monitoring of perceived and produced speech is required, and those with a higher working memory capacity also showed a higher degree of synchrony. This is in line with previous studies that suggest that working memory supports temporal information processing by encoding and maintaining the temporal intervals to be used later as a reference for synchronization and to maintain regularity (Broadway & Engle, 2011; Gibbon et al., 1984; Teki & Griffiths, 2014).

While formal musical background (number of musical instruments and years of music education) does not uniquely explain individual differences in SSST once other factors are considered, this is similar to previous studies that could not predict group affiliation based on musical training alone (Assaneo et al., 2019; Kern et al., 2021; Sjuls et al., 2023). Although other studies using the Goldsmiths Musical Sophistication index subscales (Müllensiefen et al., 2014) have found that musical training was a predictor of degree of synchrony, and music perception was a predictor only in those classified as high synchronizers, it may reflect that among trained individuals a transfer effect to perceptual abilities is more likely to be seen (Rimmele et al., 2022).

It is also worth considering the type of musical instrument played could also contribute to better sensorimotor synchronization, as it has been found that percussionists performed better compared to other musicians in a tapping task (Krause et al., 2010; Repp et al., 2013). In our study, the majority of participants were not professional musicians, nor were they generally active musicians. Our population reflects general sensorimotor synchronization skills in adulthood, where musical background as measured by the previously described variables seems not to be a predictor of spontaneous speech synchronization.

## Limitations

This study has several limitations. First, we did not use a standardized musical questionnaire such as Goldsmith (Müllensiefen et al., 2014), due to the lack of a validated Hungarian version at the time of data collection. It is suggested that future studies include a standardized music background questionnaire to further understand how musical background and exposure contribute to the individual differences in the degree of synchrony. It would also be valuable to incorporate additional measures of SMS to speech, for instance tapping tasks with speech stimuli, and measurement of linguistic abilities (e.g., syllabic discrimination) to further clarify the connections and dissociations between music and speech SMS. 

Another limitation is the modest sample size (*N* = 31), which limited statistical power to detect small effects. Accordingly, the absence of associations for tapping and musical background should be viewed as inconclusive rather than evidence of absence. The predominantly female composition (87.1%) also limits generalizability; although prior work has not consistently shown gender differences in SSST (Assaneo et al., 2019; Mares et al., 2023), we cannot rule them out, and future studies should recruit larger, gender-balanced samples to test gender differences with adequate power to detect smaller associations. Furthermore, the present findings are based on correlational analyses and therefore cannot determine causality.

Finally, the study did not include neural measures; further studies should also include neural measures of phase coherence to speech stimuli, to better understand individual differences in SSST performance.

## Conclusion

This study replicated the bimodal distribution of the Speech-to-Speech Synchronization Test (SSST) in a Hungarian-speaking population, consistent with the view that spontaneous auditory-motor synchronization to speech may be a cross-linguistic tendency. Modeling SSST as a continuous measure revealed working memory as the only reliable predictor of synchronization strength, while tapping performance and musical background showed no clear associations. These findings suggest that cognitive resources, rather than formal musical training, play a key role in spontaneous speech synchronization. The absence of significant associations with tapping performance may point to dissociations between speech and music sensorimotor synchronization (SMS), warranting further study. These results highlight the importance of considering individual differences in auditory-motor synchronization, and the complex interaction between cognitive resources, musical background, and SMS. Future research should use larger, more balanced samples, standardized musical measures, and neural indices to clarify the mechanisms underlying individual differences in SMS to speech.

## Supplementary Information

Below is the link to the electronic supplementary material.

Supplementary Material 1 (DOCX 14.6 KB)

## Data Availability

The data that support the findings of this study are available upon request to the corresponding author.
