# Supplementary material for: Linking spontaneous speech synchronization and cognitive abilities: evidence from a syllable-timed language
Source: Psychol Res. 2026 Apr 25;90(3):81. doi: 10.1007/s00426-026-02302-9 (PMC13110228; doi:10.1007/s00426-026-02302-9)
Supplement: Supplementary file 1 — Supplementary Material 1 (DOCX 14.6 KB) [file 426_2026_2302_MOESM1_ESM.docx]

Supplementary materials

Table 1

*Hungarian phonemes and the corresponding syllables used for the SSST stimuli*

| Hungarian phoneme | Syllable |
| --- | --- |
| /oː/ | bó |
| /aː/ | dá |
| /i/ | fi |
| /u/ | gu |
| /aː/ | ká |
| /u/ | lu |
| /ɛ/ | me |
| /ɛ/ | ne |
| /oː/ | nó |
| /aː/ | pá |
| /aː/ | rá |
| /u/ | tu |

The set of 12 syllables were randomly combined without gaps between them and avoiding consecutive repetition of syllables to create the stimuli of the SSST. The total duration of syllable trail was 60s.
